# Betacellulin-Induced α-Cell Proliferation Is Mediated by ErbB3 and ErbB4, and May Contribute to β-Cell Regeneration

**DOI:** 10.3389/fcell.2020.605110

**Published:** 2021-01-21

**Authors:** Young-Sun Lee, Gyun Jee Song, Hee-Sook Jun

**Affiliations:** ^1^Lee Gil Ya Cancer and Diabetes Institute, Gachon University, Incheon, South Korea; ^2^Department of Medical Science, College of Medicine, Catholic Kwandong University, Gangneung, South Korea; ^3^Translational Brain Research Center, International St. Mary's Hospital, Catholic Kwandong University, Incheon, South Korea; ^4^College of Pharmacy, Gachon University, Incheon, South Korea; ^5^Gachon Medical and Convergence Institute, Gachon Gil Medical Center, Incheon, South Korea

**Keywords:** pancreatic α-cell, pancreatic β-cell, regeneration, betacellulin, ErbB receptor

## Abstract

Betacellulin (BTC), an epidermal growth factor family, is known to promote β-cell regeneration. Recently, pancreatic α-cells have been highlighted as a source of new β-cells. We investigated the effect of BTC on α-cells. Insulin+glucagon+ double stained bihormonal cell levels and pancreatic and duodenal homeobox-1 expression were increased in mice treated with recombinant adenovirus-expressing BTC (rAd-BTC) and β-cell-ablated islet cells treated with BTC. In the islets of rAd-BTC-treated mice, both BrdU+glucagon+ and BrdU+insulin+ cell levels were significantly increased, with BrdU+glucagon+ cells showing the greater increase. Treatment of αTC1-9 cells with BTC significantly increased proliferation and cyclin D2 expression. BTC induced phosphorylation of ErbB receptors in αTC1-9 cells. The proliferative effect of BTC was mediated by ErbB-3 or ErbB-4 receptor kinase. BTC increased phosphorylation of ERK1/2, AKT, and mTOR and PC1/3 expression and GLP-1 production in α-cells, but BTC-induced proliferation was not changed by the GLP-1 receptor antagonist, exendin-9. We suggest that BTC has a direct role in α-cell proliferation via interaction with ErbB-3 and ErbB-4 receptors, and these increased α-cells might be a source of new β-cells.

## Introduction

Diabetes mellitus (DM) is characterized by prolonged high blood glucose levels due to absolute or relative deficiency of insulin. One of the beneficial strategies postulated as a possible therapy for diabetes is to increase functional insulin-producing β-cells via endogenous pancreatic β-cell regeneration. The pancreatic β-cell regeneration could occur through promotion of existing β-cell replication or conversion of other cells into β-cell (Thorel et al., 2010; Aguayo-Mazzucato and Bonner-Weir, 2018).

Pancreatic islets are organized into glucagon-producing α-cells, insulin-secreting β-cells, somatostatin-releasing δ-cells, pancreatic polypeptide-producing PP-cells, and ghrelin-secreting ε-cells (Baetens et al., 1977; Berts et al., 1997; Wierup et al., 2014; Briant et al., 2016). The pancreatic β-cells produce insulin, which stimulates glucose uptake by muscle, adipose tissue, and liver, contributing to the lowering of blood glucose levels. The pancreatic α-cells are known to play an important role in blood glucose homeostasis through production of glucagon, which stimulates hepatic glucose production. However, recent reports indicated that the possible conversion of non-β-cells in the islets to β-cells to replenish the reduced β-cell mass is possible (Thorel et al., 2010; Aguayo-Mazzucato and Bonner-Weir, 2018; Gromada et al., 2018). In this regard, α-cells have been highlighted in the islets as a source of new β-cells and a direct progenitor of β-cells under conditions of extreme destruction of β-cells (Thorel et al., 2010).

Betacellulin (BTC), a member of the epidermal growth factor (EGF) family, was originally isolated from mouse beta cell tumor (betaTC-3) as a growth–promoting factor and is known to be expressed in pancreatic α-cells, β-cells, and duct cells in adult humans (Miyagawa et al., 1999). BTC acts through binding to four ErbB tyrosine kinase receptors (EGF-R/ErbB-1, neu/ErbB-2, ErbB-3, and ErbB-4) (Wieduwilt and Moasser, 2008). It was reported that EGF-R-deficiency in mice impairs pancreatic islet development (Miettinen et al., 2000). In addition, BTC is known to promote β-cell proliferation and insulin secretion and BTC induces neogenesis of β-cells and increases the number of islet-like cell clusters, consisting primarily of β-cells (Yamamoto et al., 2000). Several studies have also shown the regenerative effects of BTC on β-cells to ameliorate diabetes (Li et al., 2003; Song et al., 2014).

We have previously found that administration of recombinant adenovirus expressing BTC improved hyperglycemia by promoting regeneration of β-cells in streptozotocin-induced diabetic mice and that β-cell proliferation was one of the mechanisms for the regeneration of β-cells by BTC (Shin et al., 2008). However, the effects of BTC on α-cells remains uncharacterized. In this study, we investigated the possible contribution of BTC on β-cell regeneration via α-cell proliferation and the mechanisms involved in α-cell proliferation by BTC.

## Materials and Methods

### Animals

C57BL/6 mice were obtained from the Korea Research Institute of Bioscience and Biotechnology (Daejeon, Korea). These mice were maintained at the facility at Gachon University under a 12 h light:12 h dark photoperiod. Animals were fed *ad libitum* on a standard rodent diet. All animal experiments were carried out under a protocol approved by the Institutional Animal Care and Use Committee at Lee Gil Ya Cancer and Diabetes Institute, Gachon University.

### Production of Recombinant Adenovirus Producing BTC

Recombinant adenovirus (rAd) producing BTC (rAd-BTC) and the control, rAd producing β-galactosidase (rAd-βgal) were constructed and produced, as previously reported (Shin et al., 2008). The recombinant adenoviruses were amplified in a human embryonic kidney cell line (HEK-293). After purification of virus by CsCl-gradient ultracentrifugation, the viral titer was determined by 50% tissue culture infectious dose (TCID50).

### Streptozotocin, rAd-BTC and BrdU Treatment

β-cell destruction was achieved in C57BL/6 mice by i.p. injection of STZ (Sigma, 150 mg/kg), a β-cell specific toxin. The mice were monitored for the development of hyperglycemia using a glucometer. STZ-induced diabetic mice (blood glucose levels > 300 mg/dl for 3 consecutive days) were injected via the tail vein with rAd-BTC or rAd-βgal with 3 × 10^9^ plaque-forming units (pfu). After viral injection, C57BL/6 mice were injected with 5-bromodeoxyuridine (BrdU; Sigma, 100 mg/kg) every day for 4 weeks.

### Intraperitoneal Glucose Tolerance Tests

Mice were not fed for 4 h and a 20% glucose solution was injected intraperitoneally (2 g/kg body weight). Blood glucose levels were measured at 0, 30, 60, 90, and 120 min following glucose injection.

### Immunohistochemical Analysis

C57BL/6 mice were sacrificed at 4 weeks after rAd-BTC or rAd-βgal injection. Pancreata were removed, fixed in 10% formalin, and embedded in paraffin. More than 200 serial sections (4 μm thick) were prepared from each pancreas, and every 20–25th section was used for immunohistochemical analysis. The tissue sections were boiled (100°C for 10 min, 10 mM sodium citrate, pH 6.0) for antigen retrieval, and blocked with blocking solution (DAKO, Carpinteria, CA, USA). The sections were then incubated with primary antibody solution: guinea-pig anti-insulin (DAKO, 1:100), rabbit anti-glucagon (DAKO, 1:100), mouse anti-PDX-1 (DSHB, Iowa, IA, 1:100) or mouse anti-BrdU (DAKO, 1:50). Fluorescein isothiocyanate (FITC)-conjugated goat anti-rabbit IgG (Santa Cruz Biotechnology, 1:200), Texas Red (TR)-conjugated goat anti-mouse IgG (Santa Cruz Biotechnology, 1:200) or Alexa-Fluor-633-conjugated goat anti-guinea-pig IgG (Thermo Fisher Scientific, Rockford, IL, 1:200) were used as secondary antibodies. Fluorescence was imaged using a laser scanning confocal fluorescent microscope (LSM 700, Carl Zeiss MicroImaging, Jena, Germany) and colocalization was analyzed using the ZEN 2009 Analysis Program.

### Islet Isolation and Immunocytochemistry

Pancreatic islets were isolated from 8 to 10-week-old male C57BL/6 mice as previously described (Jun et al., 1999). Intact islets were dissociated at 37°C in Accutase (Millipore), given STZ (1 mM) for 15 h, washed with fresh media, and then cultured with BTC (1 nM). The islet cells or the αTC1-9 cells were fixed in 4% paraformaldehyde, permeabilized in permeabilization buffer (Thermo Fisher Scientific), blocked in blocking solution (Thermo Fisher Scientific), and then incubated with mouse anti-glucagon (Sigma, 1:100), rabbit anti-glucagon (DAKO, 1:100), mouse anti-PDX-1 (DSHB, Iowa, IA, 1:100) or mouse anti-BrdU (DAKO, 1:50) antibodies. FITC-conjugated goat anti-mouse IgG (Santa Cruz Biotechnology, 1:200) or TR-conjugated goat anti-mouse IgG or anti-rabbit IgG (Santa Cruz Biotechnology, 1:200) were used as secondary antibodies. Fluorescence was imaged using a laser scanning confocal fluorescent microscope (LSM 700).

### αTC1-9 Cell Culture Conditions

The αTC1 clone 9 (αTC1-9) was obtained from American Type Culture Collection (ATCC, CRL-2350). This is a pancreatic α-cell line that produces glucagon, but not preproinsulin mRNA. αTC1-9 were cultured in Dulbecco's modified Eagle medium (DMEM) containing 16.7 mM glucose supplemented with 10% heat-inactivated dialyzed fetal bovine serum, 15 mM HEPES, 0.1 mM non-essential amino acids, and 0.02% BSA under an atmosphere of 95% humidified air-5% CO_2_ at 37°C.

### RT-PCR and Real-Time Quantitative PCR

RT-PCR was performed under the following conditions: ErbB-1 and ErbB-2: 35 cycles of 1 min at 94°C, 1 min at 60°C and 1 min at 72°C. ErbB-3 and ErbB-4: 35 cycles of 1 min at 94°C, 1 min at 58°C and 1 min at 72°C. Real-time quantitative PCR was performed using SYBR master mix (Applied Biosystems, Carlsbad, CA, USA) and carried out in a 7900HT fast real-time PCR system (Applied Biosystems). The specific PCR primers are given in Supplementary Table 1. The relative copy number was calculated using the threshold crossing point (Ct) as calculated by the 7900HT fast real time PCR software combined with the delta-delta Ct calculations.

### Proliferation Assays

αTC1-9 cells were seeded in 96-well plates at a density of 2 × 10^4^ cells (100 μL) /well and cultured with or without BTC (0, 0.25, 0.5, 1 nM) for 24 h or 48 h (BTC was added every 24 h). The cells were pulsed with [^3^H]-thymidine (1 μCi/well). Eight hours after [^3^H]-thymidine addition, the cells were analyzed for [^3^H]-thymidine incorporation using a scintillation β-counter, 1450 LSC & Luminescence Counter MicroBeta TriLux (Perkin Elmer). αTC1-9 cells were seeded in 96-well plates at a density of 2 × 10^4^ cells (100 μL) /well and cultured with BTC (1 nM) and with or without ErbB-1 inhibitor: AG1478 (4 nM) (Calbiochem, La Jolla, CA, USA), ErbB-2 inhibitor: AG825 (4 nM) (Calbiochem), ErbB-1,-2 and−3 inhibitor: AZD8931 (8 nM) (Selleckchem, Houston, TX, USA, IC50 = 4, 3, and 4 nM), ErbB-1,-2 and−4 inhibitor: AST 1306 tosylate (1.6 nM) (Selleckchem, IC50 = 0.5, 3.0, and 0.8 nM) for 24 or 48 h. Cell proliferation was measured by incubating with 10 μl of D-Plus ^TM^ CCK reagent (Dongjin LS, Seoul, Korea) for 2 h at 37°C. The optical density at 450 nm was determined and control cells were considered to represent 100%.

### Western Blotting

Whole lysates of cells were prepared as previously described. Western blotting was performed with rabbit-anti-cyclin D2 (Cell Signaling Technology), mouse-anti-cyclin E (Cell Signaling Technology), rabbit-cyclin A2 (NOVUS Biologicals), mouse-cyclin D3 (Cell Signaling Technology), mouse-anti-β actin (Santa Cruz Biotechnology), rabbit-anti-ErbB-1 (Santa Cruz Biotechnology), rabbit-anti-ErbB-2 (Santa Cruz Biotechnology), rabbit-anti-ErbB-3 (Santa Cruz Biotechnology), mouse-anti-ErbB-4 (Santa Cruz Biotechnology), mouse-anti-phospho-Tyr (Millipore), rabbit-anti-phospho-Erk1/2 (Cell Signaling Technology), rabbit-anti-Erk1/2 (Cell Signaling Technology), rabbit-anti-phospho-Akt (Ser473) (Cell Signaling Technology), rabbit-anti-Akt (Cell Signaling Technology), rabbit-anti-phospho-mTOR (Cell Signaling Technology), rabbit-anti-mTOR (Cell Signaling Technology), or mouse-anti-GAPDH (Santa Cruz Biotechnology).

### GLP-1 (7-36) Analysis

αTC1-9 cells were treated with BTC (0, 0.5, 1, or 2 nM) for 24 h. Active GLP-1 (7-36) secretion was analyzed with cultured media. Rat islets were isolated and STZ (1 mM) was added for 15 h to ablate the β-cells. The STZ-treated islets were given new media and treated with BTC (1 nM added for 24 h) for 48 h. Active GLP-1 (7–36) secretion was measured and expressed as a percentage of the STZ-treated islet value. GLP-1 (7–36) secretion was analyzed by enzyme-linked immunosorbent assay (ELISA) (R&D System), with values normalized to protein.

### Statistical Analysis

Data are presented as means ± SD or ± SE. Statistical analysis was performed using an unpaired parametric Student's *t*-test for two groups or ANOVA followed by Fisher's protected least significant difference test for multiple groups. *P* < 0.05 was accepted as significant.

## Results

### Bihormonal Cells (Insulin^+^Glucagon^+^) Are Increased in rAd-BTC-Injected Mice

To confirm that rAd-BTC treatment efficiently regulates blood glucose levels, we injected rAd-BTC or rAd-βgal (4 × 10^9^ pfu) into STZ-induced diabetic C57BL/6 male mice and blood glucose levels were monitored for 4 weeks. Blood glucose levels in rAd-BTC-treated mice were significantly decreased, compared with those in rAd-βgal-treated mice (Supplementary Figure 1A). Intraperitoneal glucose tolerance tests at 2 weeks after rAd-BTC treatment showed that blood glucose levels in rAd-BTC-treated mice were significantly lower at the 60, 90, and 120 min points following glucose injection compared with the rAd-βgal-treated mice (Supplementary Figure 1B), indicating that rAd-BTC treatment showed the glucose-lowering effects, as we previously found (Shin et al., 2008).

**Figure 1:**
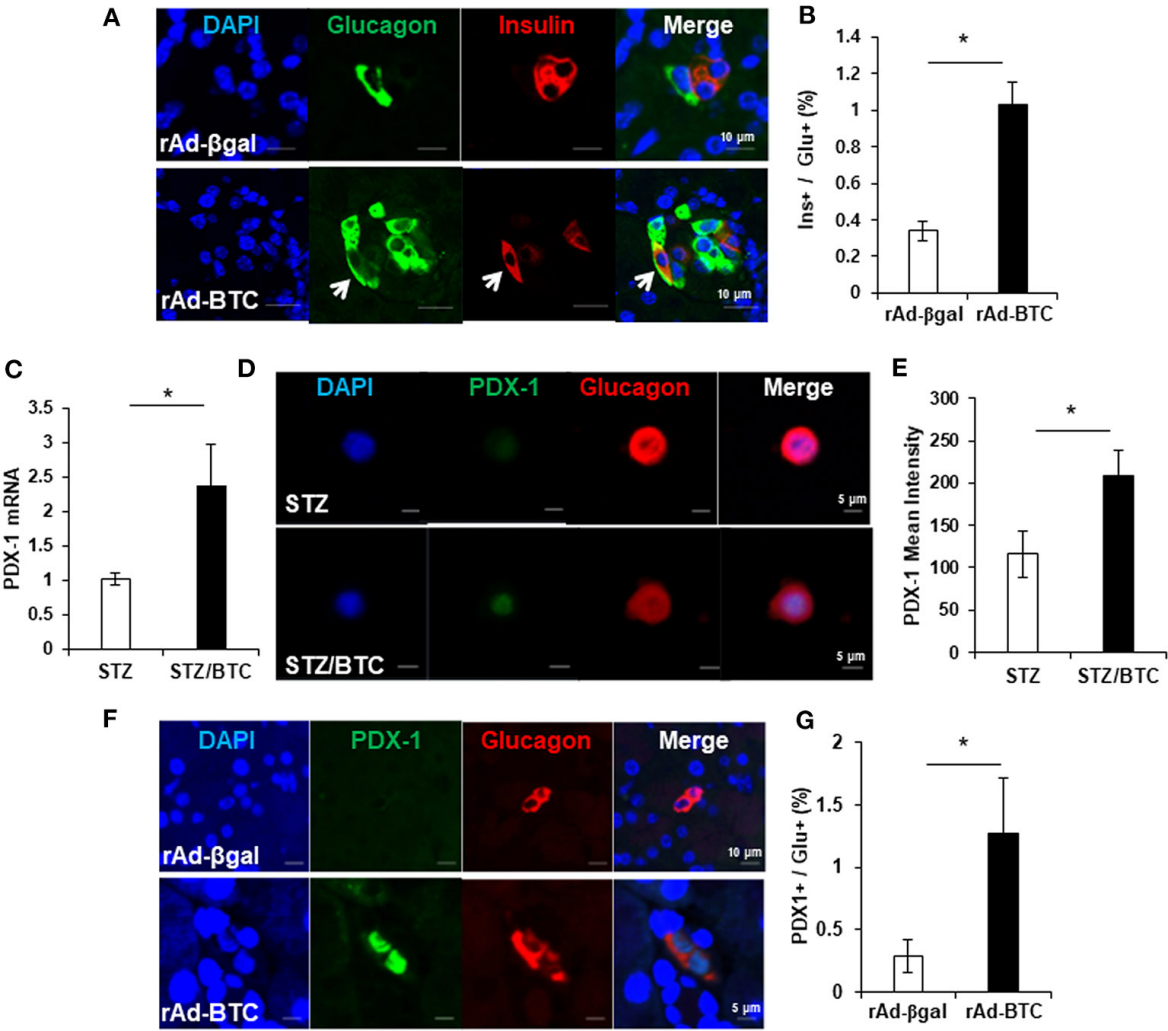
Increase of insulin+glucagon+ bihormonal cells in rAd-BTC-injected C57BL/6 mice. STZ-induced diabetic mice were treated with rAd-BTC (black squares, *n* = 5) or rAd-βgal (white squares, *n* = 4). **(A)** At 4 weeks after rAd-BTC or rAd-βgal virus injection, pancreatic sections were stained with anti-glucagon (Glu) and anti-insulin (Ins) antibodies. **(B)** The bihormonal (ins+glu+) cells were counted and expressed as a percentage of the number of glucagon+ cells in rAd-BTC or rAd-βgal injected mice (*n* = 92 or 40 islet). **(C)** Islet cells prepared form C57BL/6 mice (islet number = 400~450/group) were given STZ (1 mM) for 15 h followed by BTC (1 nM) for 24 or 48 h (add per 24 h). After 24 h, the expression of PDX-1 mRNA was analyzed by real-time quantitative PCR and normalized by cyclophilin expression. **(D)** After 48 h, the islet cells were double-stained with anti-PDX-1 and anti-glucagon antibodies. **(E)** The mean intensity was measured by confocal laser scanning microscope LSM 700 (Carl Zeiss) and analyzed with STZ- or STZ/BTC-treated islet cells (*n* = 13 or 17). **(F)** At 4 weeks after rAd-BTC virus injection, pancreatic sections were stained with anti-PDX-1 and anti-glucagon (Glu) antibodies. **(G)** The glucagon+ PDX-1+ cells were counted and expressed as a percentage of the number of glucagon+ cells in rAd-BTC or rAd-βgal injected mice (*n* = 54 or 101 islet). Data are represented as means ± SE. *P < 0.05 compared with rAd-βgal-treated mice or STZ treated cell.

Recent studies show plasticity between pancreatic α- and β-cells. α-cells can be converted into new β-cells via a bihormonal insulin+glucagon+ transitional state in animals (Parker et al., 2002; Thorel et al., 2010; Habener and Stanojevic, 2012). We therefore examined the population of bihormonal insulin+glucagon+ cells in rAd-BTC-treated mice. Immunostaining of pancreatic sections from rAd-BTC- or rAd-βgal-treated C57BL/6 mice with anti-glucagon and anti-insulin antibodies revealed that levels of insulin+glucagon+ double stained bihormonal cells were higher in rAd-BTC-treated mice than in rAd-βgal-treated mice (Figures 1A,B). To investigate whether BTC induces the expression of β-cell transcription factors such as PDX-1 in α-cells, isolated islets from C57BL/6 mice were treated with STZ to ablate β-cells, which were incubated with BTC for 24 h, and then PDX-1 mRNA expression was analyzed. We found that PDX-1 mRNA expression was significantly increased by BTC treatment (Figure 1C). In addition, immunocytochemical analysis showed that glucagon+ PDX-1+ double positive cells were observed after BTC treatment. The intensity of PDX-1 staining in glucagon-producing α-cells was significantly increased in BTC-treated cells compared with cells without BTC treatment (Figures 1D,E). To investigate whether BTC induces the expression of PDX-1 in α-cells of rAd-BTC-treated mice, the pancreatic sections from rAd-BTC- or rAd-βgal-treated C57BL/6 mice were immunostained with anti-PDX-1 and anti-glucagon antibodies. The levels of glucagon+PDX-1+ double stained cells in glucagon-producing α-cells were higher in rAd-BTC-treated mice than in rAd-βgal-treated mice (Figures 1F,G).

### Proliferation of α-Cells Is Increased in rAd-BTC-Injected Mice and in BTC-Treated αTC1-9 Cells

**Figure 2:**
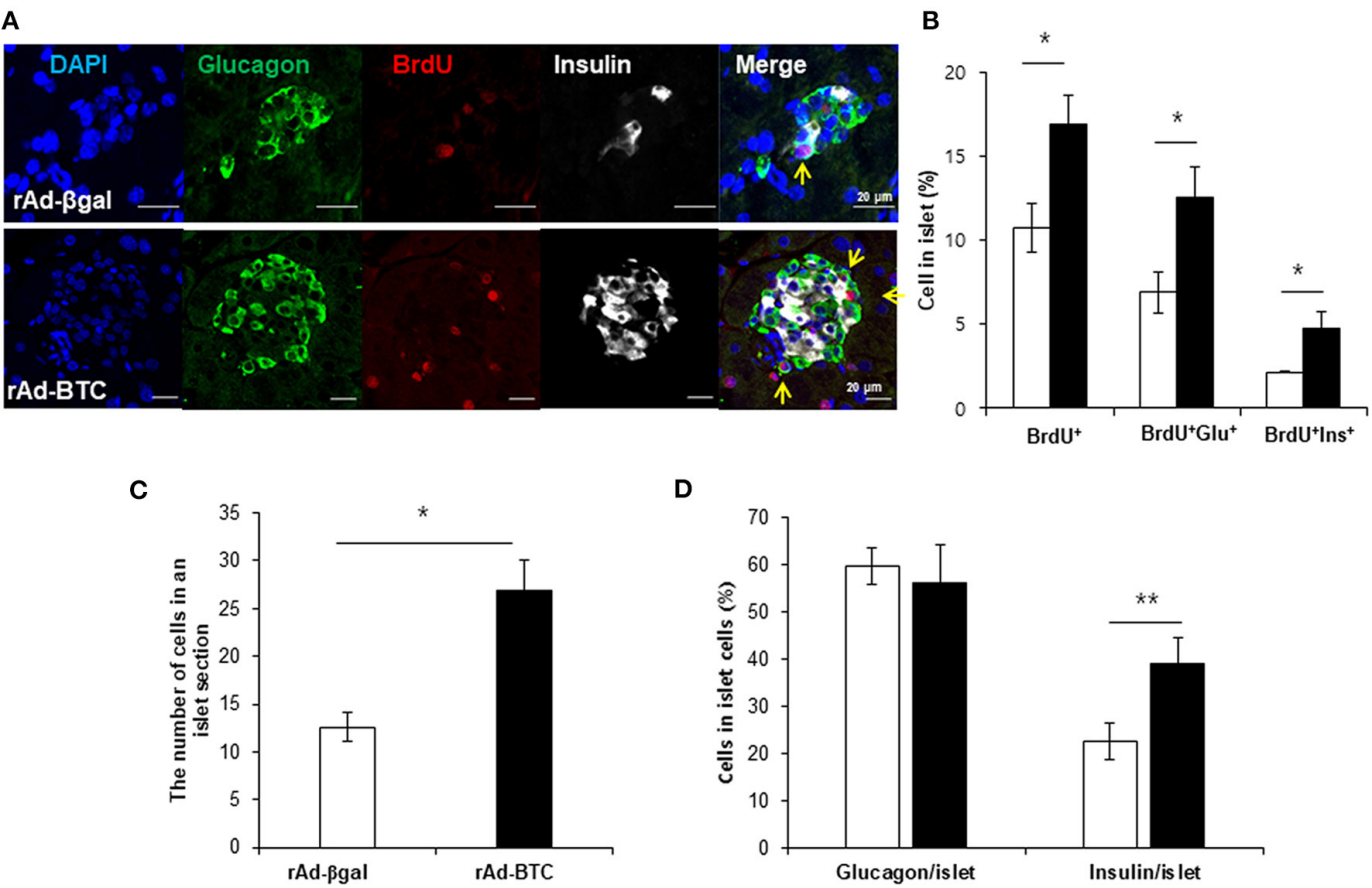
Proliferation of α-cells is increased in rAd-BTC-injected mice. STZ-induced diabetic C57BL/6 mice were injected with rAd-BTC (black, *n* = 3) or rAd-βgal (white, *n* = 3). Mice were then injected with BrdU (100 mg/kg, i.p) for 4 weeks, beginning on the first day following virus injection. **(A)** At 4 weeks after virus injection, pancreatic sections were stained with anti-glucagon (Glu), anti-insulin (Ins), and anti-BrdU antibodies. **(B)** The total BrdU+, BrdU+Glu+, and BrdU+Ins+ cell levels were calculated as a percentage of the number of total islet cells in rAd-BTC or rAd-βgal injected mice (*n* = 105 or 69 islet). **(C)** The cell number in an islet section were calculated as an average of the cell number per islet in rAd-BTC or rAd-βgal injected mice (*n* = 101 or 23 islet). **(D)** The Glu+ or Ins+ in islet cells were calculated as a percentage of the number of total islet cells in rAd-BTC or rAd-βgal injected mice (*n* = 105 or 56 islet). Data are means ± SE. *P < 0.05, **P < 0.01 compared with rAd-βgal-treated mice.

Since insulin+glucagon+ bihormonal cells were increased by BTC, we hypothesized that BTC may increase the proliferation of α-cells and these increased α-cells may contribute to β-cells regeneration. To determine the validity of this, we first determined which cells are proliferated in rAd-BTC-treated mice by injecting BrdU (daily, i.p) for 4 weeks. BrdU-positive cells in the islets were significantly increased in rAd-BTC-treated mice compared with the rAd-βgal-treated mice. BrdU+insulin+ cells were significantly increased in the islets of rAd-BTC-treated mice compared with rAd-βgal-treated mice, as expected. Interestingly, BrdU+glucagon+ cells showed a greater increase than BrdU+insulin+ cells (Figures 2A,B). To investigate whether the cell numbers in an islet section was increased by BTC treatment, we counted the cell number in the islets. The average cell number in an islet was significantly increased in rAd-BTC-treated mice compared with rAd-βgal-treated mice (Figure 2C). In addition, we calculated the pancreatic α- or β-cell proportion. We found that the pancreatic α-cell proportion was similar between STZ-rAd-βgal-treated mice and STZ-rAd-BTC-treated mice. However, the pancreatic β-cell proportion was significantly increased in rAd-BTC-administered mice, even though the proliferation of α-cells was increased in STZ-rAd-BTC-treated mice (Figure 2D).

**Figure 3:**
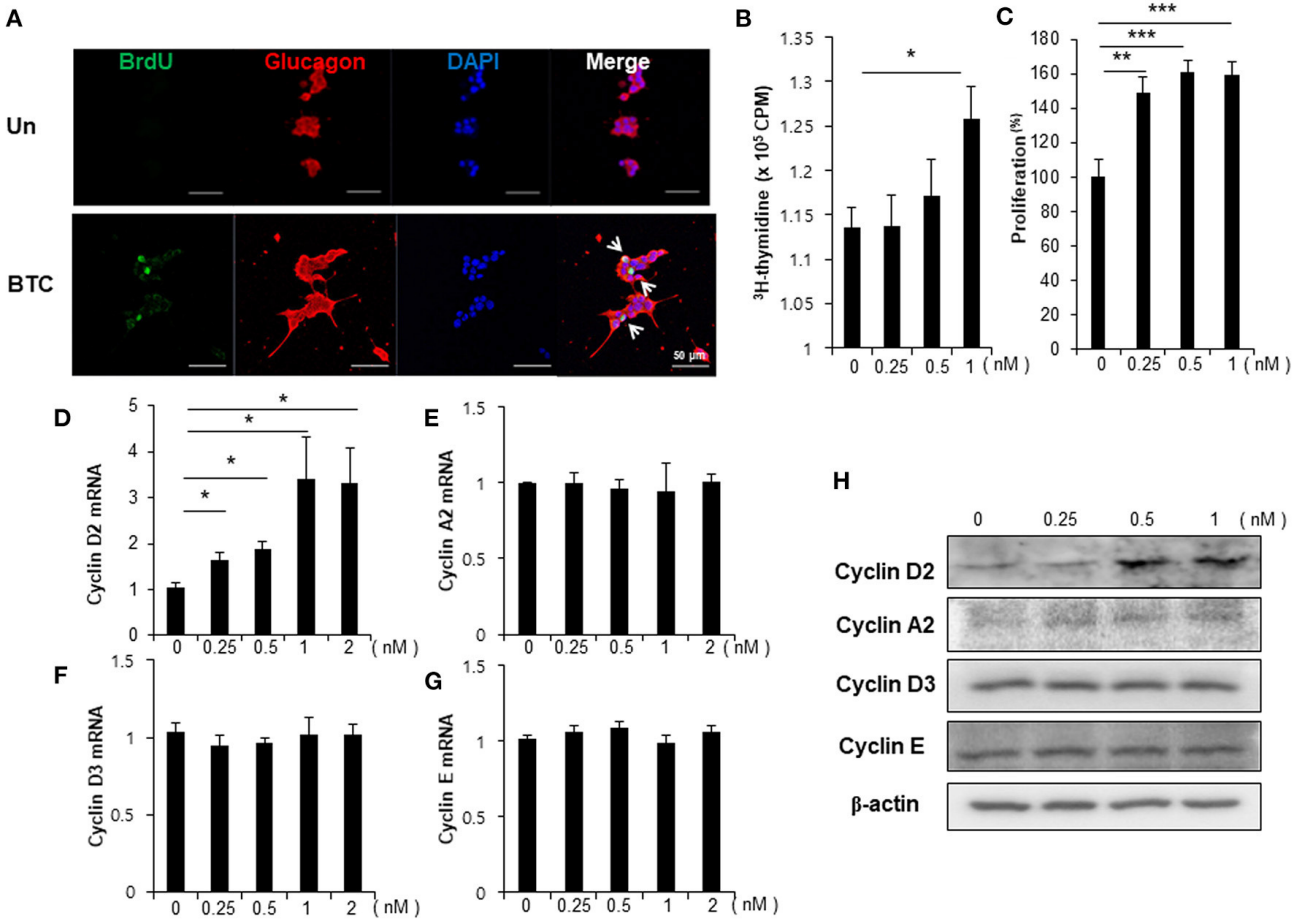
Proliferation of α-cells is increased in BTC-treated αTC1-9 cells. αTC1-9 cells were cultured without (Un) or with BTC (1 nM) for 2 days. **(A)** αTC1-9 cells were pulsed with BrdU for 24 h, and cells were then double-stained with anti-BrdU and anti-glucagon antibodies. **(B)** αTC1-9 cells were pulsed with [^3^H]-thymidine for 6 h and [^3^H]-thymidine incorporation was measured. **(C)** αTC1-9 cells were incubated with BTC (1 nM) for 24 h. Viable cells were measured by CCK8 assay. The data are presented as a percentage of the untreated control. The expression of **(D)** cyclin D2, **(E)** cyclin A2, **(F)** cyclin D3, **(G)** cyclin E mRNA was analyzed by real-time quantitative PCR, and **(H)** the proteins were analyzed by western blotting. Data are means ± SE from three to four independent experiments and are expressed as a ratio of the control **(D–G)**. *P < 0.05, **P < 0.005, ***P < 0.0005 compared with untreated cells.

To examine whether BTC signaling directly increases proliferation in α-cells, αTC1-9 cells were cultured in the presence of BrdU. We found that BrdU incorporated α-cells were increased in BTC-treated αTC1-9 cells (Figure 3A). [^3^H]-thymidine incorporation assays and CCK8 proliferation assays also showed that BTC significantly increased proliferation of αTC1-9 cells in a dose dependent manner (Figures 3B,C). When we analyzed the expression of cyclins, which are important proteins that control the proliferation of cells through the cell cycle, the expression of cyclin D2 mRNA and protein was significantly increased; however, the expression of cyclin A2, cyclin E, and cyclin D3 was not changed in BTC-treated αTC1-9 cells (Figures 3D–H).

### ErbBs Are Activated in αTC1-9 Cells by BTC-Treatment

**Figure 4:**
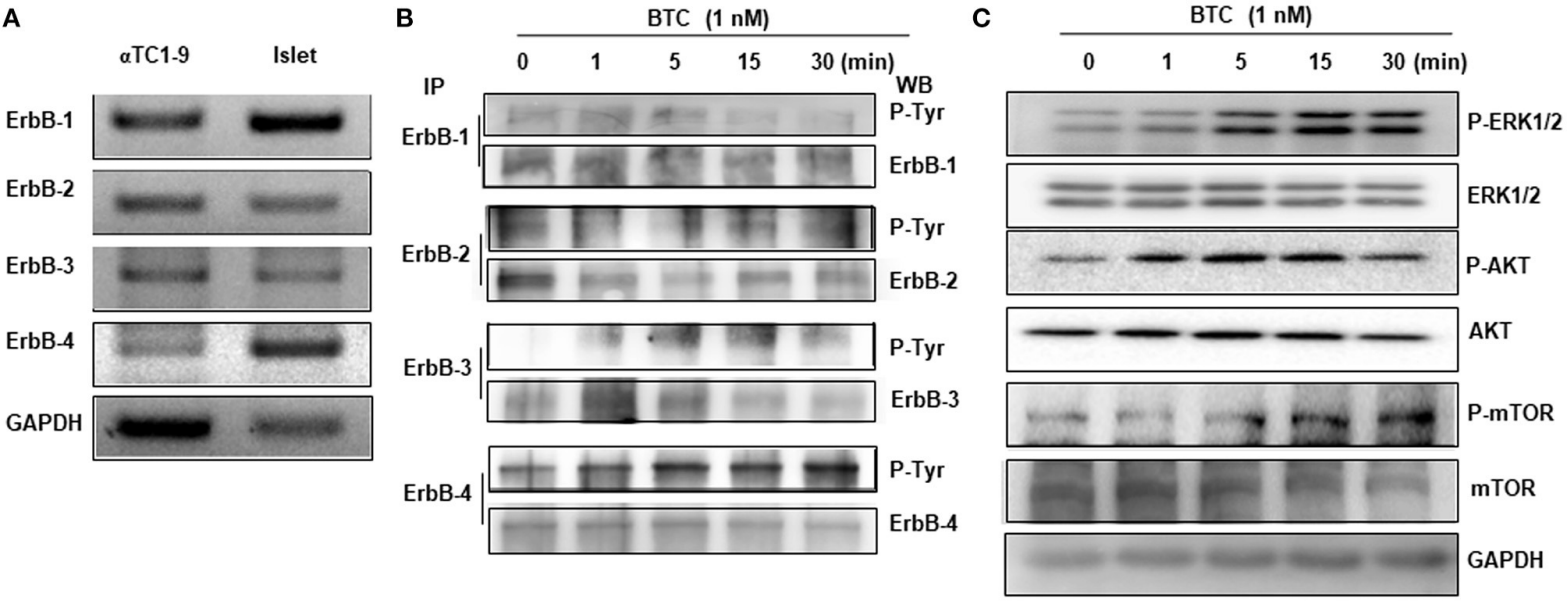
Expression and phosphorylation of ErbBs in BTC-treated αTC1-9 cells. **(A)** RT-PCR analysis of the expression of ErbB-1,−2,−3, and−4 in αTC1-9 cells and isolated C57BL/6 mouse islets. **(B)** αTC1-9 cells were treated with BTC for the indicated time. Cells were harvested and cell lysate was immunoprecipitated (IP) with anti-ErbB antibodies and then detected by phosphorylated ErbBs by western blot using phospho-Tyr antibody. **(C)** p-ERK1/2, p-AKT (Ser473), and p-mTOR, as well as ERK1/2, AKT, and mTOR were measured by western blot of total cell extracts. Results are representative of three independent experiments.

As BTC binds and activates ErbB receptors, we first examined the expression of ErbB receptors in αTC1-9 cells. The expression of ErbB-1, ErbB-2, ErbB-3, and ErbB-4 mRNA was detected in αTC1-9 cells, and islets from C57BL/6 mice also expressed all ErbB receptors (Figure 4A).

We then investigated which ErbB receptors are activated by BTC treatment. αTC1-9 cells were treated with 1 nM BTC for 0, 1, 5, 15, and 30 min and then phosphorylation of ErbB receptors was determined by immunoprecipitation and western blotting. Phosphorylation was observed in ErbB-1, ErbB-2, ErbB-3, and ErbB-4 following BTC treatment in αTC1-9 cells (Figure 4B). Activation of ErbB induces the stimulation of intracellular pathways such as Ras/Raf/mitogen-activated protein kinase (MAPK)/ extracellular signal-regulated kinase (ERK) (MEK)/ERK, and phosphatidylinositol-3-kinase (PI3K)/AKT/mammalian target of rapamycin (mTOR) (Arteaga and Engelman, 2014). Thus, we examined the activation of ERK1/2, AKT, and mTOR and found that the phosphorylation of ERK, AKT, and mTOR was also increased after BTC treatment in αTC1-9 cells (Figure 4C).

### BTC-Induced Proliferation of αTC1-9 Cells Is Mediated by ErbB-3 and ErbB-4

**Figure 5:**
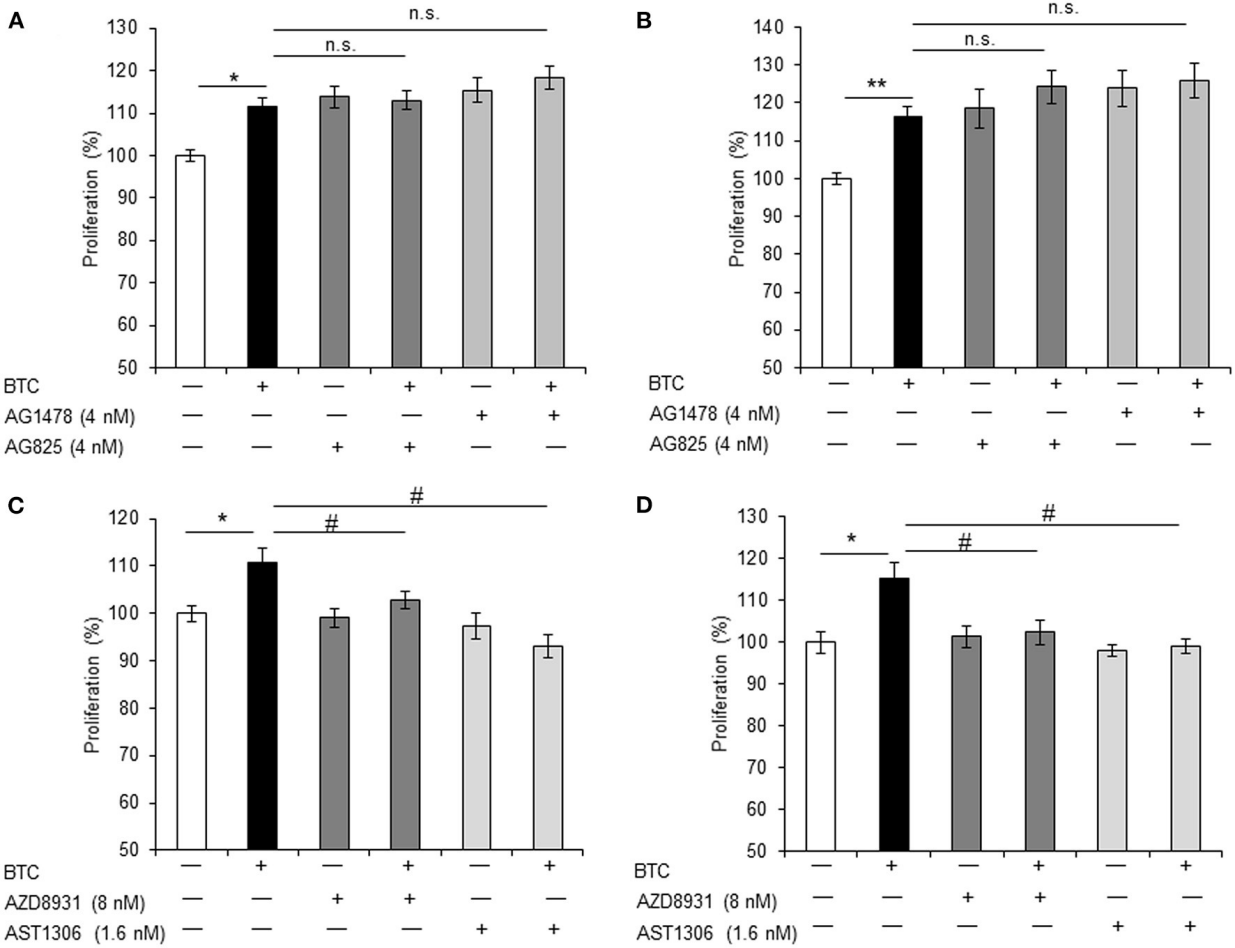
BTC-induced α-cell proliferation is inhibited by ErbB-3 and ErbB-4 inhibitors. αTC1-9 cells were pretreated with ErbB-1 inhibitor: AG1478, ErbB-2 inhibitor: AG825, ErbB-1,-2 and−3 inhibitor: AZD8931, or ErbB-1,-2 and−4 inhibitor: AST 1306 tosylate for 30 min and BTC (1 nM) was then added and incubated for 24 h **(A,C)** or 48 h **(B,D)**. Viable cells were measured by CCK8 assay. The data are presented as a percentage of the untreated control. Data are means ± SE from three to four independent experiments. *P < 0.05, **P < 0.01 compared with untreated cells, ^#^*P* < 0.05, n.s. (not significant) compared with BTC treated cells.

Because all ErbBs (ErbB-1, ErbB-2, ErbB-3, and ErbB-4) were activated by BTC treatment in αTC1-9 cells, we investigated which ErbBs are involved in BTC-induced proliferation. αTC1-9 cells were stimulated with BTC (1 nM) for 24 h and 48 h in the presence of 4 nM of AG1478 or AG825 as specific ErbB-1 or ErbB-2 inhibitor. In BTC treated αTC1-9 cells, proliferation was significantly increased and specific ErbB-1 and ErbB-2 inhibitors did not show inhibitory effects on BTC-induced proliferation (Figures 5A,B).

Treatment with either AZD8931 (inhibition of ErbB-1, ErbB-2, and ErbB3) or AST1306 tosylate (inhibition of ErbB-1, ErbB-2 and ErbB4), significantly suppressed BTC-induced proliferation of αTC1-9 cells (Figures 5C,D). These results suggest that BTC increases α-cell proliferation via activation of ErbB-3 and ErbB-4 receptors in αTC1-9 cells, not by ErbB-1 and ErbB-2 receptors.

### BTC-Induced αTC1-9 Cell Proliferation Is Independent of GLP-1 Secretion by BTC

**Figure 6:**
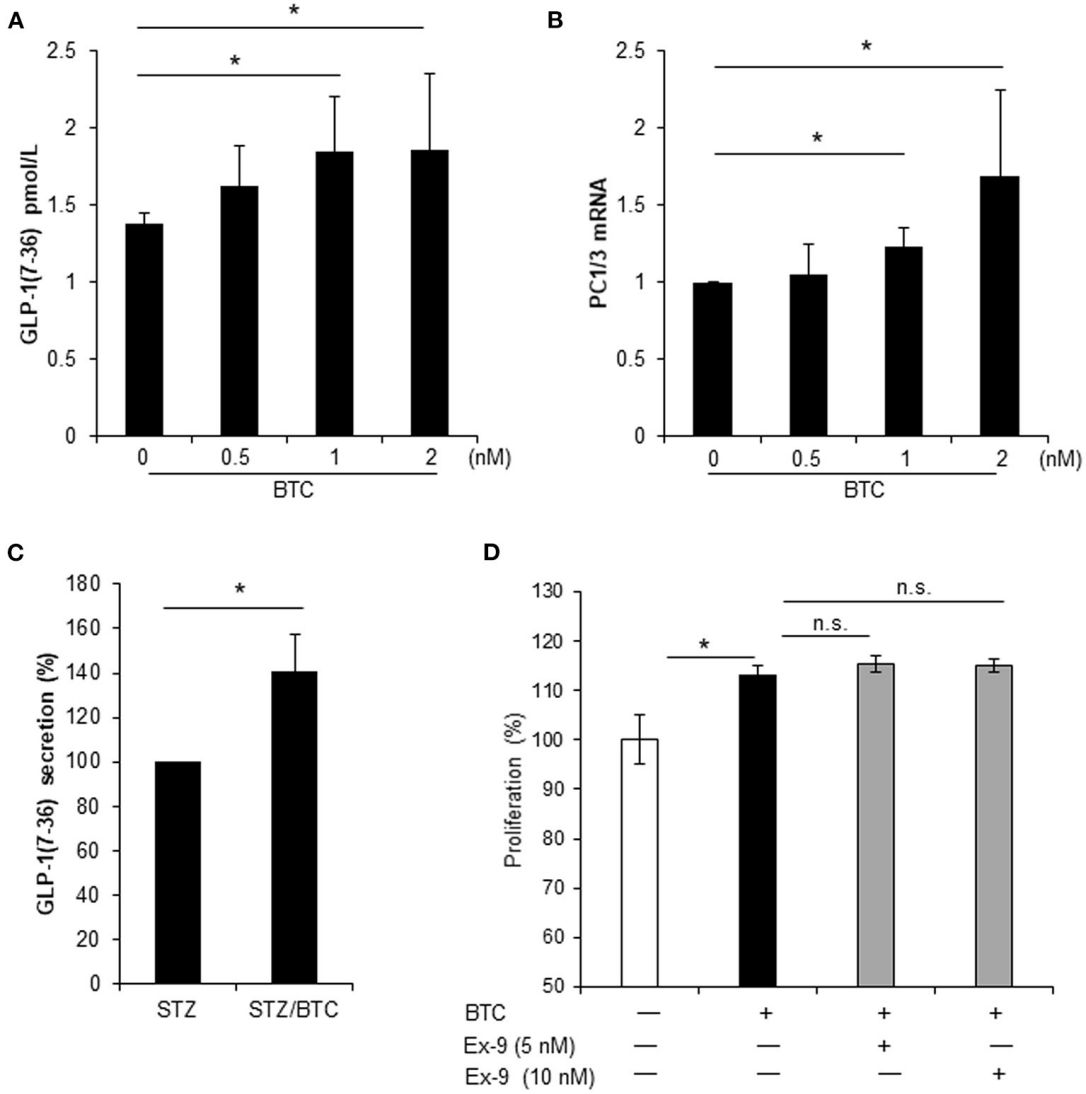
BTC increased GLP-1(7-36) secretion in αTC1-9 cells and rat islets but BTC-induced GLP-1 did not affect cell proliferation. **(A)** αTC1-9 cells were treated with the indicated concentration of BTC for 24 h and GLP-1 (active 7-36) secretion in media was measured. **(B)** αTC1-9 cells were treated with the indicated concentration of BTC for 6 h and the expression of PC1/3 mRNA was measured by RT-qPCR. **(C)** Rat islets were isolated and treated with STZ (1 mM) for 15 h and then with or without BTC (1 nM) for 48 h (add per 24 h). Active GLP-1 secretion in media was measured and expressed as a percentage of the value of STZ-treated islet. **(D)** αTC1-9 cells were pretreated with exendin-9 (5 nM or 10 nM) for 30 min and BTC was then added, incubated for 24 h, and a CCK8 assay was performed (n = 6~12). Data are expressed as a percentage of the value of untreated cells. Data are means ± SE from three to four independent experiments. *P < 0.05 compared with untreated or STZ only-treated islets and n.s. (not significant) compared with BTC treated cells.

Local secretion of GLP-1 from pancreatic α-cells may be beneficial for β-cell function and β-cell regeneration (Masur et al., 2005; Chen et al., 2012; Lee et al., 2018). Thus, we examined whether BTC induces GLP-1 secretion in α-cells. BTC induces GLP-1 secretion in αTC1-9 cells in a dose dependent manner at 24 h after BTC treatment (Figure 6A). GLP-1 is produced by PC1/3 in pancreatic α-cells (Masur et al., 2005; Chen et al., 2012). Thus, we determined the expression of PC1/3 mRNA in BTC treated αTC1-9 cells. We found that BTC treatment increased PC1/3 expression at 12 h after BTC treatment (Figure 6B). In addition, BTC treatment also significantly increased GLP-1 secretion in β-cell ablated islets following STZ treatment (mainly α-cells remain) (Figure 6C). To investigate whether BTC-induced proliferation in α-cells is mediated by the increased GLP-1 secretion by BTC, we pretreated αTC1-9 cells with a GLP-1 receptor antagonist, exendin-9, and then treated with BTC. However, BTC-induced proliferation was not changed by exendin-9 (Figure 6D) in αTC1-9 cells, suggesting that BTC increased pancreatic α-cell proliferation by itself, not mediated by GLP-1.

## Discussion

Pancreatic α-cells are endocrine cells in the islets of the pancreas. The α-cells synthesize proglucagon which is cleaved by prohormone convertase 2 (PC2) to produce active glucagon. Glucagon is secreted by α-cells in response to hypoglycemia. Glucagon is a counter-regulatory hormone to insulin which together maintain homeostasis of glucose metabolism, which is the classical role of pancreatic α-cells. However, recent reports indicate an emerging role for pancreatic α-cells, as a source of newly generated β-cells (Gromada et al., 2018; Lee et al., 2018). Transdifferentiation of α-cells to β-cells was observed under conditions of extreme physiological demand for insulin (Thorel et al., 2010). The number of pancreatic α-cells increased under conditions of insufficient insulin, due to damage to β-cells, as found in patients of recent-onset type 1 diabetes (Yabe et al., 2013) and STZ-induced diabetic animals (Dusaulcy et al., 2016). In addition, the number of pancreatic α-cells also increased under the insulin resistance condition of high fat diet-induced obesity (Ellingsgaard et al., 2008). However, the reasons for compensatory increase of pancreatic α-cells are not yet clear.

We previously found that rAd-BTC treatment regenerated pancreatic β-cells and lowered blood glucose levels in STZ-induced diabetic mice, and BTC-induced β-cell proliferation was one of the mechanisms for β-cell regeneration (Shin et al., 2008). In this study, we investigated the effect of BTC on the proliferation of α-cells and found that α-cell proliferation increased in the pancreas of rAd-BTC-treated mice compared with rAd-βgal-treated mice after destruction of β-cells with STZ. In addition, we found that the proliferation of αTC1-9, a pancreatic α-cell line, increased with BTC treatment.

An increase of α-cell proliferation might increase glucagon production, which can increase blood glucose levels through hepatic glucose output, and deteriorates the diabetic condition. However, rAd-BTC did not increase blood glucagon levels (Supplementary Figure 2). We found that BTC induces GLP-1 secretion in αTC1-9 and β-cell-ablated islets (Figures 6A–C). GLP-1 has glucose-lowering effects as it inhibits glucagon secretion and stimulates insulin secretion in islets (Kieffer and Habener, 1999; Drucker, 2006; Holst et al., 2011). Thus, we speculate that BTC-induced GLP-1 secretion might inhibit glucagon secretion, contributing to the attenuation of hyperglycemia, even despite the increase in proliferation of α-cells.

Recent studies show that insulin + glucagon + bihormonal cells can become new-β cells in animals with almost total destruction of β-cells (Thorel et al., 2010). In this study, BTC increased not only pancreatic α-cells, but also insulin+glucagon+ bihormonal cells following rAd-BTC treatment in STZ-induced diabetic mice (Figures 1A,B). In addition, BTC increased the expression of PDX-1, an important transcription factor for the differentiation of progenitor cells into the β-cell phenotype, in α-cells and BTC has been known to have effects in regulation of β-cell mass (Shin et al., 2008; Oh et al., 2015). Therefore, it is possible to generate new β-cells via insulin+glucagon+ bihormonal cells and induction of PDX-1. The expression of PDX-1 mRNA was significantly increased at 24 h after BTC treatment in β-cell ablated islet cells (Figure 1C). In mice, PDX-1 is expressed during E7.5 −11.5 and re-expressed after E13.5 (Zhu et al., 2017) and forced PDX-1 expression induces α-cells to β-cells conversion (Yang et al., 2011). In this study, the levels of glucagon+PDX-1+ double stained cells in glucagon-producing cells were higher in rAd-BTC-treated mice than in rAd-βgal-treated mice (Figures 1F,G). Therefore, BTC induced PDX-1 might contribute to transdifferentiation of α-cells to β-cells. However, we need to further study for lineage tracing experiment, using for example gcg-rtTA;TetOcre;R-26YFP mice, where α-cells are tagged with YFP. In addition, the somatostatin producing δ-cells were significantly increased in rAd-BTC-treated mice compared with the rAd-βgal-treated group (Supplementary Figure 3), however we couldn't observe somatostatin + insulin + bihormonal cells. We need to further study for the effect of BTC on δ-cells.

BTC is a member of the EGF family and a ligand for the four tyrosine kinase receptors (ErbB-1/EGFR, neu/ErbB-2/HER2, ErbB-3/HER3 and ErbB-4/HER4). These receptors can form homo- and heterodimers with one another (Graus-Porta et al., 1997; Wieduwilt and Moasser, 2008). It was reported that BTC binds ErbB-1 and ErbB-3 heterodimers (Rush et al., 2018) or ErbB-1 and ErbB-4 heterodimers (Gomez-Gaviro et al., 2012). BTC was shown to induce proliferation of pancreatic β-cells through the activation of ErbB-1 and ErbB-2 receptors (Oh et al., 2011). We found that all ErbBs (ErbB-1, ErbB-2, ErbB-3, and ErbB-4) were expressed in both αTC1-9 cells and in islets of C57BL/6 mice. BTC-induced α-cell proliferation was not affected by specific inhibitors of ErbB-1 or ErbB2, but inhibited by pan-ErbB inhibitors AZD8931 (inhibition of ErbB-1, ErbB-2 and ErbB3) or AST 1306 tosylate (inhibition of ErbB-1, ErbB-2 and ErbB4), suggesting that BTC may induce proliferation of pancreatic α-cells via the ErbB-3 and ErbB-4 receptor. However, the role of each ErbB receptors in BTC-induced α-cell proliferation needs further study.

ErbB receptors contribute to biological effects through the activation of PI3K/AKT/mTOR and MAPK/ERK signaling pathways (Kainulainen et al., 2000; Mishra et al., 2018). mTOR regulates cell growth, cell proliferation, and cell survival (Lipton and Sahin, 2014) and its function is mediated through mTOR complex 1 (mTORC1) and mTOR complex 2 (mTORC2). The loss of mTORC1 signaling in α-cells reduced α-cell mass, which resulted from decreased proliferation and increased apoptosis during postnatal maintenance (Bozadjieva et al., 2017). In this study, BTC increased mTOR phosphorylation in αTC1-9 cells, which might regulate α-cell proliferation. In addition, BTC enhanced growth and migration of vascular smooth muscle cells through activation of ERK1/2, AKT, and MAPK (Mifune et al., 2004). We also found increased phosphorylation of ERK1/2 and AKT in BTC-treated αTC1-9 cells. These results indicate that activation of PI3K/AKT/mTOR and MAPK/ERK signaling pathways might be the mechanisms underlying BTC-induced α-cell proliferation.

Pancreatic α-cells produce GLP-1 through post-translational processing of proglucagon by PC1/3 (Piro et al., 2014; Knop, 2018). Interestingly, we found that BTC also induced GLP-1 secretion and PC1/3 expression in αTC1-9 cell and β-cell ablated islets. In a previous study, we reported that treatment with GLP-1 increases the proliferation in α-cell and transdifferentiation of α-cell to β-cells, as analyzed using lineage tracing transgenic mouse models (Lee et al., 2018). Thus, we consider that GLP-1-induced by BTC can affect proliferation of α-cells. Therefore, we investigated whether the increase in GLP-1 caused by BTC treatment increases proliferation of α-cells. We used the GLP-1 receptor antagonist, exendin-9, to inhibit the effect of GLP-1 in BTC-induced α-cell proliferation. We found that BTC-induced proliferation was not changed by exendin-9 in α-cells. These results suggest that the effect of BTC induced α-cell proliferation is a direct effect of BTC on α-cell, not an indirect effect mediated by BTC-induced GLP-1. In this study, BTC increased GLP-1 secretion in BTC-treated α-cells, however this may be not enough to increase α-cell proliferation. Further studies are required to identify the detailed mechanisms.

In conclusion, we investigated the effect of BTC treatment on proliferation of pancreatic α-cells and found that treatment of BTC increased α-cell proliferation via ErbB receptors and activation of Erk1/2, AKT, and mTOR signaling pathways in α-cells. The increased α-cells may contribute to β-cells regeneration.

## Data Availability Statement

The original contributions presented in the study are included in the article/Supplementary Material, further inquiries can be directed to the corresponding author/s.

## Ethics Statement

The animal study was reviewed and approved by Institutional Animal Care and Use Committee at Lee Gil Ya Cancer and Diabetes Institute, Gachon University.

## Author Contributions

Y-SL designed the study, performed experiments and data analysis, and wrote the manuscript. GS performed experiments and wrote the revised manuscript. H-SJ designed the study, analyzed data, and wrote the manuscript. All authors contributed to the article and approved the submitted version.

## Conflict of Interest

The authors declare that the research was conducted in the absence of any commercial or financial relationships that could be construed as a potential conflict of interest.
