# Supplementary material for: Betacellulin-Induced α-Cell Proliferation Is Mediated by ErbB3 and ErbB4, and May Contribute to β-Cell Regeneration
Source: Front Cell Dev Biol. 2021 Jan 21;8:605110. doi: 10.3389/fcell.2020.605110 (PMC7859283; doi:10.3389/fcell.2020.605110)
Supplement: Supplementary file 1 [file Table_1.DOCX]

**Table S1. List of PCR primers and sequences.**

| Gene | sense | anti-sense |
| --- | --- | --- |
| cyclin D2 | 5’-CCGTCAAGAGCAGCATAACG-3’ | 5’-TGGCTTGGTCCGGATCTTC-3’ |
| cyclin A2 | 5’-AAGAGGCAGCCAGACATCACTAA-3’ | 5’-CTCAACCAGCCAGTCCACAA-3’ |
| cyclin D3 | 5’-TCCAAGCTGCGCGAAAC-3’ | 5’-GGTCCGTATAGATGCAAAGCTTCT-3’ |
| cyclin E | 5’-GTTCCGTTCGCCATGGTTAT-3’ | 5’-CCCGGAAGTGCTTGAGCTT-3’ |
| ErbB-1 | 5’-ATTCATGCGAAGACGTCACATT  -3’ | 5’-GTTCCACGAGCTCTCTCTCTTGA  -3’ |
| ErbB-2 | 5’-GCTGCCCGAAACGTGCTA  -3’ | 5’-CCGTGCCAGCCCGAA  -3’ |
| ErbB-3 | 5’-AGGCTTGTCTGGATTCTGTGGTT  -3’ | 5’- GGGATCGGGTGCAGAGAGA  -3’ |
| ErbB-4 | 5'-GGAGGCTGCTCAGGACCAA  -3' | 5'-ACGCAGGCTCCACTGTCAT  -3' |
| PC1/3 | 5'-ATTTTGGTGCTGCTGCTCTT-3' | 5'-GGAGTGCTCGTCTCAACCA-3' |
| GAPDH | 5'-TGAGCCCTTCCACAATGCCA-3' | 5'-AGTGCCAGCCTCGTCCCGTA-3' |
| PDX-1 | 5’-GAAATCCACCAAAGCTCACG-3’ | 5’-CGGGTTCCGCTGTGTAAG-3’ |
| cyclophilin | 5’-TGGAGAGCACCAAGACAGACA-3’ | 5’–TGCCGGAGTCGACAATGAT-3’ |
