# Supplementary figures and images for: Betacellulin-Induced α-Cell Proliferation Is Mediated by ErbB3 and ErbB4, and May Contribute to β-Cell Regeneration
Source: Front Cell Dev Biol. 2021 Jan 21;8:605110. doi: 10.3389/fcell.2020.605110 (PMC7859283; doi:10.3389/fcell.2020.605110)

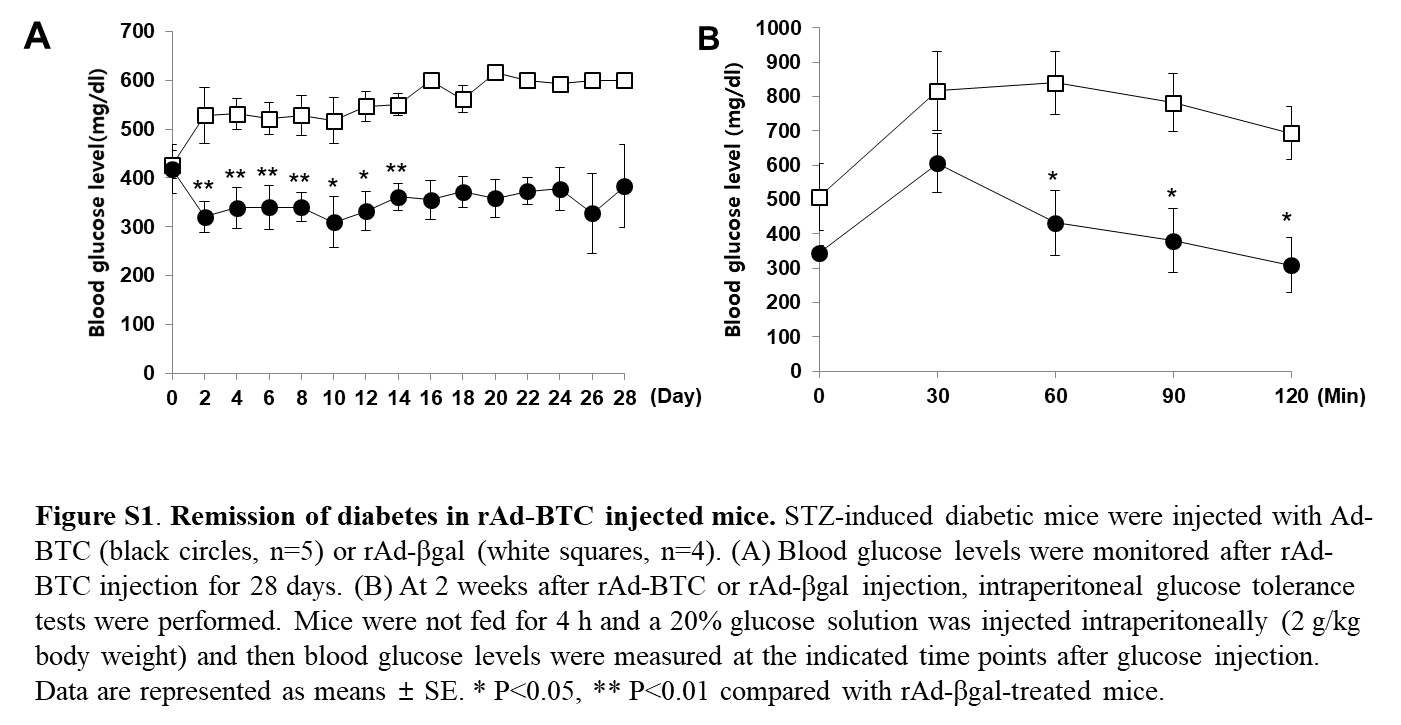


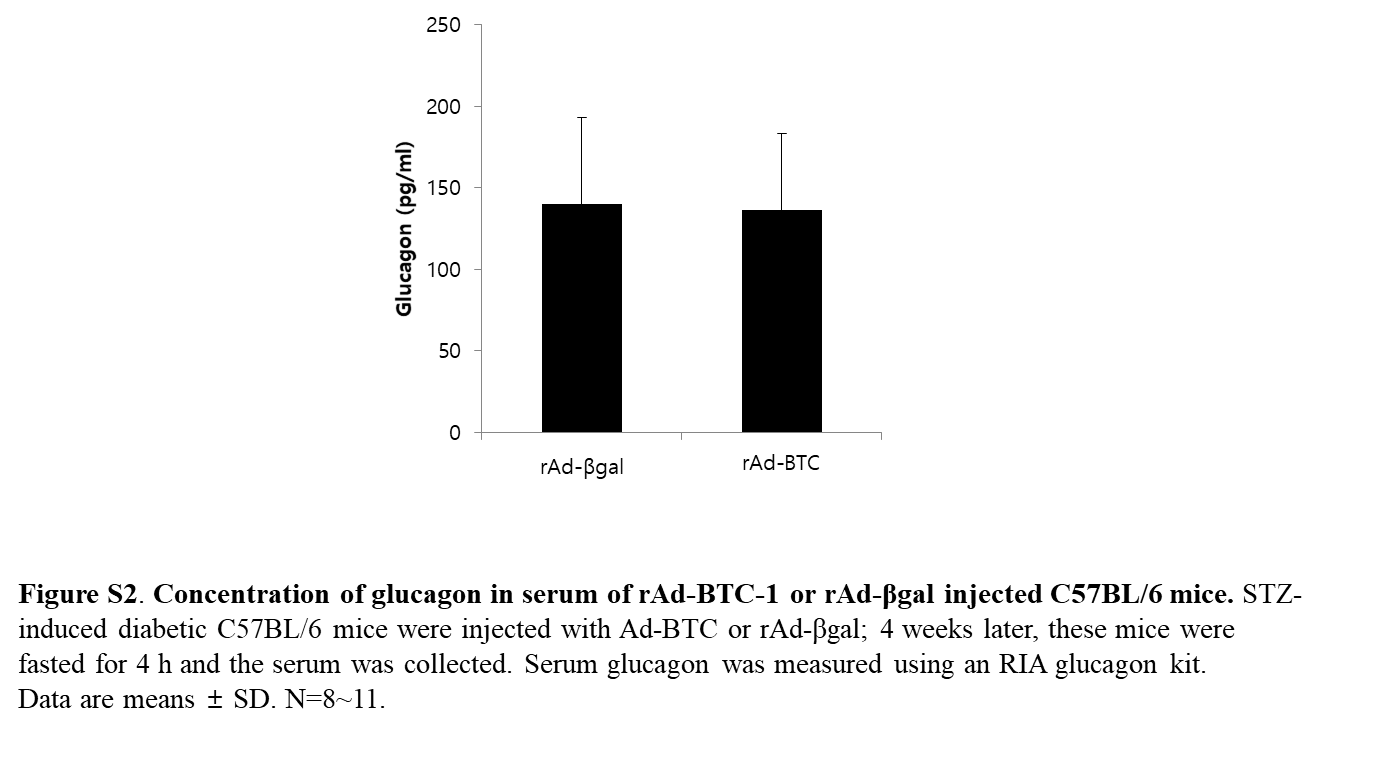


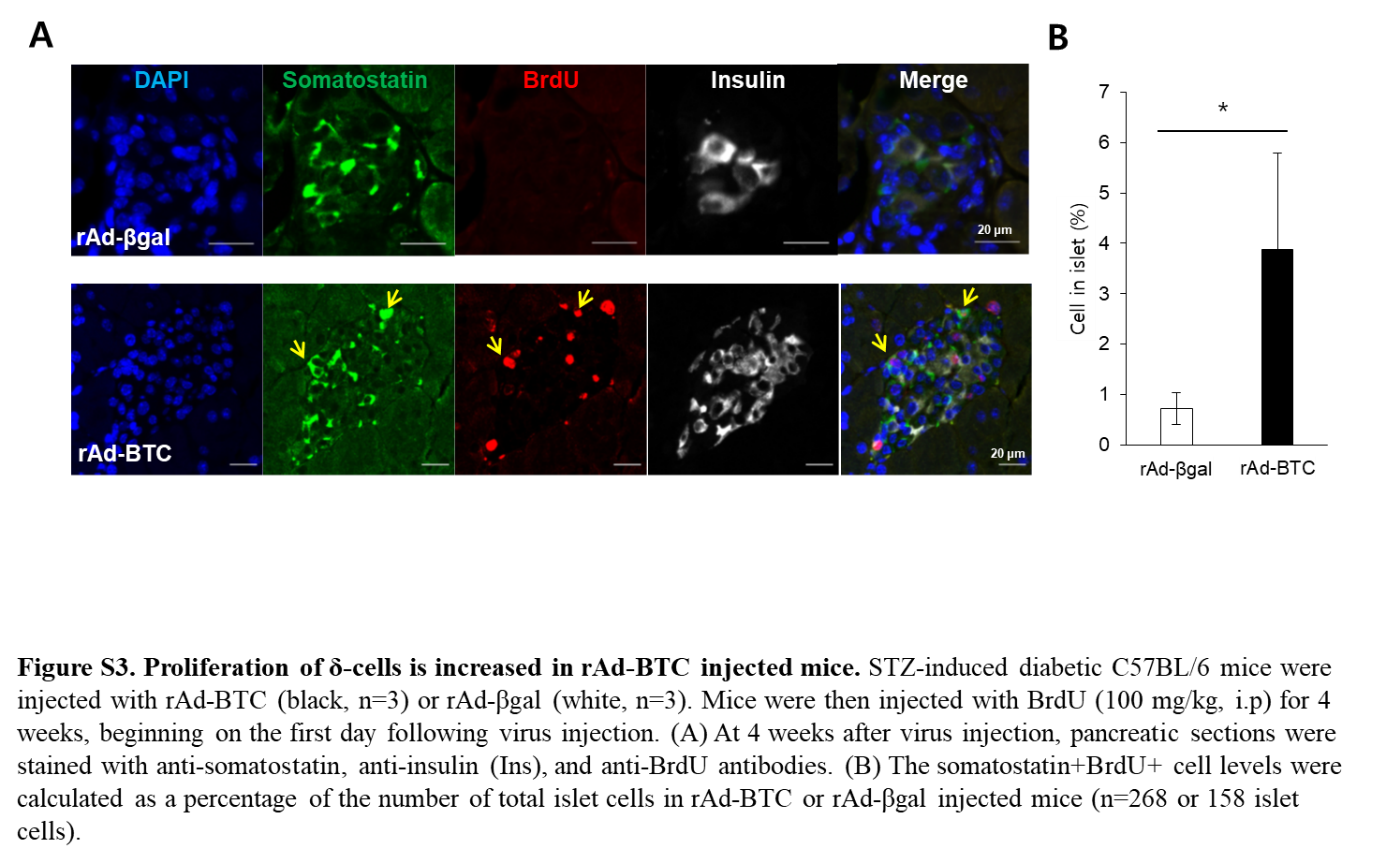

Supplement: Supplementary file 2 [file Table_2.DOCX]
